# Diagnostic performance of three-dimensional high-resolution anorectal manometry in identifying descending perineal syndrome

**DOI:** 10.3389/fmed.2026.1802595

**Published:** 2026-06-09

**Authors:** Hua Chu, You Wang, Chunrong Ma, Lijun Du

**Affiliations:** 1 Department of Gastroenterology, Sir Run Run Shaw Hospital, School of Medicine, Zhejiang University, Hangzhou, China; 2 Department of Radiology, Sir Run Run Shaw Hospital, School of Medicine, Zhejiang University, Hangzhou, China

**Keywords:** area under the curve, conventional defecography, descending perineal syndrome, dyssynergic pattern, three-dimensional high-resolution anorectal manometry

## Abstract

**Background:**

Three-dimensional high-resolution anorectal manometry (3DHRAM) has been developed and introduced to explore pelvic floor disorder. The aim of this study was to evaluate the diagnostic performance of 3DHRAM in identifying descending perineal syndrome (DPS) in patients with obstructed defecation.

**Methods:**

All consecutive patients referred in the previous 1 year for investigations of obstructed defecation were eligible. We included only patients who underwent both 3DHRAM and CD. Binary logistic regression was performed to identify independent predictors of DPS. The sensitivity, specificity, Youden index and area under ROC were calculated in order to propose a diagnostic strategy for DPS.

**Results:**

Thirty-eight patients (41.3%) presented with DPS on CD. Binary logistic regression analysis confirmed that perineal descent was independently associated with DPS (odds ratio = 1.13, 95% confidence interval 1.03–1.23, *P* = 0.009). On 3DHRAM, the excessive perineal descent parameter indicated a sensitivity of 0.61, a specificity of 0.76 with a Youden index of 0.37, and derived a cut-off value of 10.5 mm and achieved an area under the curve of 0.72. Perineal descent combined with rectocele, rectal mucosal prolapse, and dyssynergic pattern derived the best Youden Index (0.44) with a specificity of 0.89 and a sensitivity of 0.55.

**Conclusion:**

This study suggests that 3DHRAM has potential as a complementary tool for diagnosing DPS at present. Future multi-center, prospective studies with larger, more diverse cohorts are needed to validate our findings and to better define the role of 3DHRAM in diagnosing DPS.

## Introduction

Pelvic floor disorders represent a significant health concern, affecting 12% to over 20% of women (1). These disorders, which often manifest as fecal incontinence or constipation, can lead to substantial health-care costs and a markedly decreased quality of life. Descending perineal syndrome (DPS) is a challenging subtype of pelvic floor disorder that often leads to obstructed defecation. DPS is characterized by excessive bulging of perineum during straining, although perineal descent can also be seen at rest (2). DPS entails a “vicious cycle” of straining and constipation, which leads to more straining and exacerbation of the anatomical abnormality and descent.

Accurate diagnosis and effective management of DPS are therefore critical for improving patient outcomes. Currently, the diagnosis of DPS depends on magnetic resonance (MR)-defecography or conventional defecography (CD) (3). However, CD involves exposure to ionizing radiation, while MR-defecography is expensive and not widely accessible (4). Both techniques are time-consuming and are often poorly tolerated by patients, leading to low acceptability and compliance (3).

Previous research has highlighted the potential of high-resolution anorectal manometry (HRAM) in identifying defecatory disorders caused by both functional and structural abnormalities (5). The recent development of three-dimensional HRAM (3DHRAM) provides simultaneous physiological and morphological data, offers a more comprehensive assessment of the anorectal region compared to traditional manometry, introduces new diagnostic possibilities for pelvic floor disorders (6). Beyond diagnosing anorectal functional disorders, 3DHRAM can also detect anal sphincter defects (7).

The aim of this study was to evaluate the diagnostic performance of 3DHRAM in identifying DPS among patients with obstructed defecation, using CD as the reference standard. By comparing the diagnostic accuracy of 3DHRAM with CD, we sought to determine whether 3DHRAM could serve as a reliable alternative or complementary tool for diagnosing DPS.

## Materials and methods

### Patients

This single-center retrospective study included all consecutive patients referred for obstructed defecation workup between January and December 2023. Only those who underwent both 3DHRAM and CD examinations were enrolled. Inclusion criteria were: age ≥18 years, presence of obstructed defecation symptoms, and an interval ≤ 1 week between the two examinations. Exclusion criteria were: age <18 years, interval >1 week between 3DHRAM and CD, organic colorectal pathology by colonoscopy, prior pelvic floor surgery, inflammatory bowel disease, diabetes mellitus, systemic sclerosis, or chronic neurological diseases. For each patient we recorded age, gender, symptoms duration, and obstetric history. Owing to the retrospective nature of data extraction from the electronic medical system, informed consent was waived. This study was approved by the ethical committee of Sir Run Run Shaw Hospital (No. 2023-718-01).

### Conventional defecography

After rectal filling with 300 ml contrast agent, patients sat on a specially designed commode and were instructed to contract the pelvic floor musculature and then empty the rectum as completely as possible. Fluoroscopic images were recorded during several maneuvers to assess and measure the descent of the pelvic floor and to diagnose rectocele or enterocele. DPS was defined as excessive descent of the anorectal angle below the pubococcygeal line during straining: >3.5 cm in multiparous women and >3 cm in men and nulliparous women. A rectocele was defined as any anterior bulge >2 cm beyond the extrapolated line of the anterior rectal wall. The pubococcygeal (PC) line drawn from the lower border of the pubic symphysis to the last coccygeal joint is considered a good boundary of the normal pelvic floor. The H line was the line drawn from the lower end of the pubic bone to the posterior rectal wall at the level of the anorectal junction, where the puborectalis muscle was visible. The H line indicates the width of the levator hiatus. The M line is the line drawn perpendicular to the PC line from the posterior end of the H line. The M line quantified the degree of descent of the pelvic floor. Prolapse was diagnosed when the rectal mucosa extended beyond the anorectal ring on straining (8).

### Three-dimensional high-resolution anorectal manometry

**FIGURE 1:**
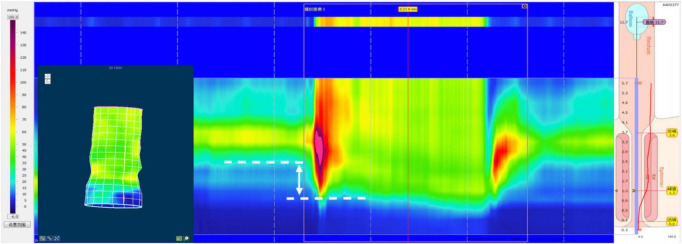
Pressure variation during a straining effort on 3DHRAM, correspond to the criterion “excessive perineal descent,” indicated by the arrow between the two dotted lines.

We used Mano Scan360 3D solid-state HRAM instrument (Medtronic, USA). Digital rectal examination was routinely performed before probe insertion. The lubricated probe was manually introduced into the anorectum and was held to prevent movements. After a 5-min resting period, we recorded: anal canal pressures at rest and during two straining series, voluntary squeeze pressure, length of the high-pressure zone, sensory thresholds (first sensation, desire to defecate, urgency to defecate), and presence of the recto-anal inhibitory reflex (RAIR). Dyssynergic patterns were classified into four subgroups based on rectal and anal pressures during straining effort (9, 10). Manometric data were analyzed using specific ManoView analysis software (Sierra Scientific Instruments, Los Angeles, CA, USA). Perineal descent measured by 3DHRAM followed the method described by Parks et al., which determines movement of the anal margin during a defecation effort (Figure 1) (2).

### Statistical analysis

Continuous variables were presented as means ± standard deviations (SD), and categorical variables as numbers and percentages. Between-group comparisons (DPS vs. non-DPS on CD) of continuous variables were performed using the Mann-Whitney test. The categorical data were compared using the chi-squared test or Fisher’s exact test. A multivariable binary logistic regression analysis was performed to identify independent predictors of DPS. The receiver operating characteristic (ROC) analyses were used to estimate the variable sensitivity and specificity to determine the area under the curve (AUC) and the Youden index value (calculated as sensitivity + specificity−1) of different manometric parameters and their combinations of 3DHRAM for differentiating DPS and non-DPS (11). Statistical significance was set at *P* <0.05.

## Results

### Patient

Between January 1 and December 31, 2023, a total of 92 patients (27 male) were enrolled. All presented with constipation, and five also reported fecal incontinence. Patients characteristics are summarized in Table 1.

**TABLE 1:** Patient characteristics and CD results.

Parameter	Value
Age (mean ± SD)	52.3 ± 15.6
Gender (male/female)	27/65
Symptoms	
Constipation (*n*/%)	92
Fecal incontinence (*n*/%)	5
Both symptoms (*n*/%)	0
Duration of symptoms (years) (mean ± SD)	7.0 ± 7.3
Conventional defecography results	
**Descending perineal syndrome**	
Number (*n*/%)	38 (41.3%)
Size (mm) (mean ± SD)	42.4 ± 7.4
Rectocele	
Number (*n*/%)	41 (44.6%)
Size (mm) (mean ± SD)	29.7 ± 8.0
M line	
Size (mm) (mean ± SD)	36.1 ± 18.0
H line	
Size (mm) (mean ± SD)	73.4 ± 11.2
Rectal mucosal prolapse (*n*/%)	36 (39.1%)
Dyssynergia (*n*/%)	21 (22.8%)

### Conventional defecography

Of the 92 patients who underwent CD, 38 (41.3%) were diagnosed with DPS. Additional findings included rectocele in 41 patients (44.6%), rectal mucosal prolapse in 36 (39.1%), and dyssynergia in 21 (22.8%). The mean width of the levator hiatus (H line) was 73.4 ± 11.2 mm, and the mean degree of descent of the pelvic floor (M line) is 36.1 ± 18.0 mm. All CD findings were presented in Table 1.

### Three-dimensional high-resolution anorectal manometry

No significant difference were observed between DPS and non-DPS patients in anal resting pressure, anal squeeze increment, propulsion (rectal pressure during straining), or residual pressure (anal pressure during straining) (*P* >0.05). However, the distribution of dyssynergic patterns differed significantly between the two groups (*P* = 0.036). DPS patients presented higher first sensation compared to non-DPS patients (*P* = 0.02), while no difference were found in desire to defecate and urgency to defecate sensation (*P* >0.05). The overall distribution of anorectal sensation showed no significant difference between groups (*P* = 0.314). Detailed manometric measurements were presented in Table 2.

**TABLE 2:** Three-dimensional high-resolution anorectal manometry (3DHRAM) results.

Parameter	With DPS	Without DPS	*P*-value
Patients (*n*) (male/female)	38	54	
Anal resting pressure (mmhg) (mean ± SD)	72.4 ± 18.0	67.8 ± 22.7	0.298
Anal squeeze increment (mmhg) (mean ± SD)	250.3 ± 70.3	236.4 ± 82.8	0.402
Propulsion (mmhg)	48.1 ± 15.8	47.4 ± 16.0	0.833
Residual pressure (mmhg) (mean ± SD)	78.4 ± 31.2	82.6 ± 35.4	0.544
First sensation (ml) (mean ± SD)	57.8 ± 25.9	46.2 ± 17.6	0.020
Desire to defecate (ml) (mean ± SD)	83.4 ± 41.1	69.3 ± 29.5	0.057
Urgency to defecate (ml) (mean ± SD)	155.3 ± 59.6	142.8 ± 57.6	0.316
Dyssynergic pattern (*n*/%)	28 (73.6%)	36 (66.7%)	0.001
Type I	7	21	
Type II	2	8	
Type III	13	5	
Type IV	6	2	
Rectal sensitivity			0.314
Hypersensitivity	15	30	
Hyposensitivity	16	17	
Normal	7	7	
Perineal descent (mm)	12.8 ± 5.9	8.5 ± 5.2	<0.001
Rectocele (*n*/%)	29 (76.3%)	40 (74.1%)	0.807
Rectal mucosal prolapse (*n*/%)	21 (55.3%)	24 (44.4%)	0.307

We performed a multivariable binary logistic regression analysis, after adjusting for rectocele, rectal mucosal prolapse, first sensation, and dyssynergic pattern, perineal descent distance remained the sole independent predictor of DPS (odds ratio = 1.13, 95% confidence interval 1.03–1.23, *P* = 0.009). Rectocele (*P* = 0.37), rectal mucosal prolapse (*p* = 0.78), first sensation (*P* = 0.09), and dyssynergic pattern (*P* = 0.09) did not retain statistical significance in the model.

**FIGURE 2:**
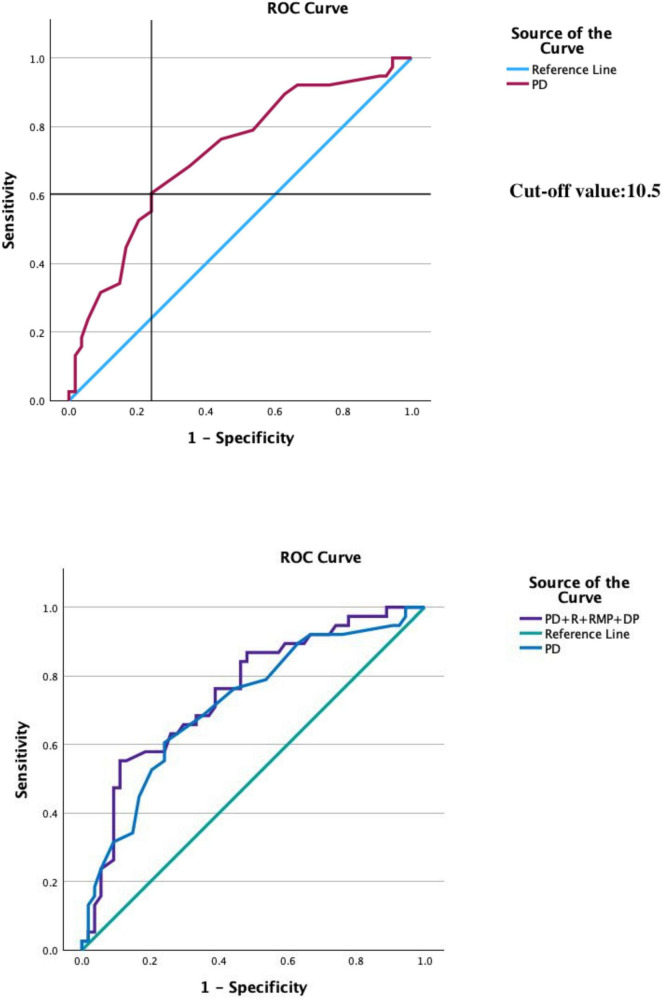
The ROC curve analysis with a cut-off value for diagnosing descending perineal syndrome based on 3DHRAM.

The perineal descent distance detected by 3DHRAM was 12.8 ± 5.9 mm in DPS group, and 8.5 ± 5.2 mm in non-DPS group (as diagnosed by CD). Using the perineal descent distance alone to diagnose DPS, yield a sensitivity of 0.61, a specificity of 0.76, a Youden index of 0.37, an AUC of 0.72, and a cut-off value of 10.5 mm. We further explored combinations of other 3DHRAM parameters to improve diagnostic performance. When perineal descent was combined with the presence of rectocele, rectal mucosal prolapse, and dyssynergic pattern, the highest Youden index of 0.44 was achieved, with a sensitivity of 0.55 and a specificity of 0.89. These data are presented in Figure 2 and Table 3.

**TABLE 3:** The diagnostic performance of 3DHRAM for DPS diagnosis according to different manometric parameters and their combinations.

Diagnostic metric	PD	PD + R	PD + RMP	PD + DP	PD + R + RMP + DP
Sensitivity	0.61	0.66	0.61	0.84	0.55
Specificity	0.76	0.72	0.76	0.56	0.89
Youden index	0.37	0.38	0.37	0.40	0.44
AUC	0.72	0.72	0.72	0.75	0.75
95% CI for AUC	0.61–0.83	0.62–0.83	0.61–0.83	0.65–0.85	0.65–0.85

PD, perineal descent; R, rectocele; RMP, rectal mucosal prolapse; DP, dyssynergic pattern; AUC, area under ROC.

## Discussion

This study represents one of the first systematic evaluations of 3DHRAM in diagnosing DPS in patients with obstructed defecation. While previous studies have suggested that morphological data obtained from 3DHRAM can help identify pelvic floor disorders, few have focused specifically on DPS (5).

Descending perineal syndrome is an uncommonly discussed condition with obstructed defecation and likely overlooked. Affected patients frequently complain of incomplete rectal evacuation followed by a sensation of obstruction. In our study, all patients reported constipation, and five also experienced fecal incontinence.

A meta-analysis reported excessive perineal descent on CD in 44.4% (range 36.2–52.7) of patients referred for chronic constipation (12). Another observational study found an even higher frequency of 85% (13). In our study, CD identified DPS in 41.3% of patients.

Notably, the absolute perineal descent values measured by 3DHRAM with a mean value of 12.8 mm were consistently lower than those obtained by CD with 42.4 mm. This discrepancy is likely explained by well-known difference in patients positioning between the two techniques. CD is performed in a seated position, which more closely mimics natural defecation and allows greater perineal descent under gravity and increased intra-abdominal pressure. While 3DHRAM is conducted in the left lateral decubitus position, a non-physiological posture that tends to underestimate perineal movement. Although previous study has suggested that the sensitivity for detecting perineal descent is better in the sitting position than in the supine position, the difference may not reach statistical significance (3). Nevertheless, the positional disparity remains a critical biomechanical factor that directly affects the absolute values we obtained, and it limits direct comparison of measurements between the two methods. Despite these differences, our study found that DPS examined by 3DHRAM achieved an AUC of 0.72, meeting the threshold for moderate diagnostic utility. Diagnostic accuracy is paramount for DPS because it directly influences treatment decisions. Since DPS is a benign condition, high specificity seems more important than high sensitivity. In our study, 3DHRAM yielded a Youden Index (0.37) with a specificity of 0.76 and a sensitivity of 0.61. Given the moderate accuracy, 3DHRAM should not be used as a standalone diagnostic test for DPS currently. Nevertheless, multivariable analysis identified perineal descent distance as an independent predictor of DPS.

Another notable finding is the distinct distribution of dyssynergic patterns between DPS and non-DPS patients. Overall, 69.5% of 92 patients exhibited one of the four dyssynergic patterns on manometry. The distribution differed significantly between groups, patients with DPS mainly presented as type III dyssynergy, while those without DPS mainly presented as type I. The ability of 3DHRAM to identify dyssynergic patterns adds to its diagnostic utility, as it provides both functional and morphological insights into the underlying causes of obstructive defecation.

In our study, DPS patients did not show elevated propulsion pressure or residual pressure compared with non-DPS patients, suggesting that the pathophysiology of DPS is multifactorial. Previous research has linked risk factors for perineal descent to rectocele and rectal intussusception (14). The finding that perineal descent remained an independent predictor supports its inclusion in diagnostic consideration. We further combined other parameters of 3DHRAM to improve diagnostic performance. The most relevant diagnostic criterion was observed when perineal descent distance combined rectocele, rectal mucosal prolapse, and dyssynergic pattern, yielding the best Youden Index of 0.44, a specificity of 0.89, but a sensitivity of only 0.55, meaning that nearly half of DPS cases would be missed. Thus, 3DHRAM is not suitable as a replacement for defecography at present. Instead, it could serve as an initial, radiation-free, office-based triage tool. A positive result of perineal descent ≥10.5 mm raises suspicion of DPS, rectocele, rectal mucosal prolapse, or a dyssynergic pattern was sequentially assessed and would justify referral for confirmatory imaging. The main value of 3DHRAM lies in providing concurrent functional and morphological information, helping prioritize patients for more extensive radiological work-up. Previous study supported our observation that functional and structural causes of obstructed defecation often overlap, and a single test of manometry or defecography may not be sufficient (15). The need for combined structural and functional assessment is further supported by recent evidence from Camur et al. that increased puborectalis muscle thickness (>4.8 mm) and abdominal subcutaneous adipose tissue thickness (>23 mm) were significantly associated with dyssynergic defecation (16). Their findings demonstrated that structural parameters, beyond functional manometric measurements, played an important role in the pathophysiology of evacuation disorders.

The limitations of this study should be acknowledged. First, this was a retrospective, single-center study, which inevitably carries selection bias. Because we only included patients who underwent both 3DHRAM and CD, our cohort likely represents a selected subgroup with more severe or refractory symptoms. Therefore, our findings may not fully generalize to broader or primary care populations. Second, the modest sample size limited statistical power. Third, we did not perform inter- or intra-observer reproducibility analyses for either technique, which is a gap given the subjective nature of some measurements. Fourth, the positional difference between 3DHRAM (lateral) and CD (sitting) is a major confounder that we could not eliminate, which likely contributed to the lower perineal descent values on 3DHRAM. Finally, we used CD as reference standard, MR-defecography offers better soft-tissue resolution and no radiation, and future studies should compare 3DHRAM against MR-defecography. These limitations call for multi-center, prospective studies with larger, more diverse cohorts to validate our findings and to better define the role of 3DHRAM in diagnosing DPS.

In conclusion, this study suggests that 3DHRAM has potential as a complementary tool for diagnosing DPS, but its moderate accuracy and low sensitivity preclude its use as a standalone test. Interpretation of 3DHRAM findings should be undertaken with caution and may include both functional and morphological parameters. At present, 3DHRAM should be used only as part of a comprehensive diagnostic strategy, ideally in combination with imaging. Although our study proposes a novel application of the morphological capabilities of 3DHRAM, further research is needed to establish new classifications and reference values for anatomic pelvic floor disorders.

## Data Availability

The original contributions presented in this study are included in this article/supplementary material, further inquiries can be directed to the corresponding author.
